# 2-Fluoro-*N*′-[(2-hydroxynaphthalen-1-yl)methylidene]benzohydrazide

**DOI:** 10.1107/S1600536811050896

**Published:** 2011-12-03

**Authors:** Dong-Yue Wang, Xu-Feng Meng, Jing-Jun Ma

**Affiliations:** a Hebei Key Laboratory of Bioinorganic Chemistry, College of Sciences, Agricultural University of Hebei, Baoding 071001, People’s Republic of China

## Abstract

In the title mol­ecule, C_18_H_13_FN_2_O_2_, the hy­droxy group is involved in an intra­molecular O—H⋯N hydrogen bond. The naphthyl ring system and the benzene ring form a dihedral angle of 31.0 (2)°. In the crystal, N—H⋯O hydrogen bonds link the mol­ecules into chains propagating in [101].

## Related literature

For the biological activity of benzohydrazide compounds, see: El-Sayed *et al.* (2011 ▶); Horiuchi *et al.* (2009 ▶). For coordination compounds with benzohydrazide ligands, see: El-Dissouky *et al.* (2010 ▶); Zhang *et al.* (2010 ▶). For standard bond lengths, see: Allen *et al.* (1987 ▶). For the crystal structures of similar compounds, see: Suleiman Gwaram *et al.* (2010 ▶); Liu *et al.* (2011 ▶); Zhou *et al.* (2011 ▶); Meng *et al.* (2011 ▶).
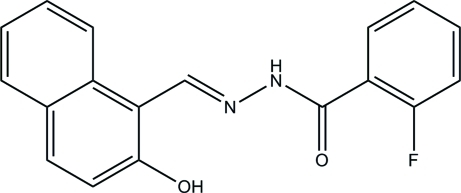


## Experimental

### 

#### Crystal data

C_18_H_13_FN_2_O_2_
*M* *_r_* = 308.30
Monoclinic, 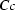

*a* = 7.078 (3) Å
*b* = 29.1953 (16) Å
*c* = 7.3013 (10) Å
β = 106.521 (3)°
*V* = 1446.5 (6) Å^3^
*Z* = 4
Mo *K*α radiation
μ = 0.10 mm^−1^
*T* = 298 K
0.20 × 0.17 × 0.17 mm

#### Data collection

Bruker SMART 1K CCD area-detector diffractometer
Absorption correction: multi-scan (*SADABS*; Sheldrick, 1996 ▶) *T*_min_ = 0.980, *T*_max_ = 0.983
4745 measured reflections
2641 independent reflections
1777 reflections with *I* > 2σ(*I*)
*R*_int_ = 0.036

#### Refinement

*R*[*F* ^2^ > 2σ(*F* ^2^)] = 0.046
*wR*(*F* ^2^) = 0.133
*S* = 1.03
2641 reflections
212 parameters
3 restraints
H atoms treated by a mixture of independent and constrained refinement
Δρ_max_ = 0.18 e Å^−3^
Δρ_min_ = −0.16 e Å^−3^

### 

Data collection: *SMART* (Bruker, 2007 ▶); cell refinement: *SAINT* (Bruker, 2007 ▶); data reduction: *SAINT*; program(s) used to solve structure: *SHELXS97* (Sheldrick, 2008 ▶); program(s) used to refine structure: *SHELXL97* (Sheldrick, 2008 ▶); molecular graphics: *SHELXTL* (Sheldrick, 2008 ▶); software used to prepare material for publication: *SHELXTL*.

## Supplementary Material

Crystal structure: contains datablock(s) I, global. DOI: 10.1107/S1600536811050896/cv5206sup1.cif

Structure factors: contains datablock(s) I. DOI: 10.1107/S1600536811050896/cv5206Isup2.hkl

Supplementary material file. DOI: 10.1107/S1600536811050896/cv5206Isup3.cml

Additional supplementary materials:  crystallographic information; 3D view; checkCIF report

## Figures and Tables

**Table 1:** Hydrogen-bond geometry (Å, °)

*D*—H⋯*A*	*D*—H	H⋯*A*	*D*⋯*A*	*D*—H⋯*A*
N1—H1⋯O1^i^	0.91 (1)	1.91 (2)	2.785 (3)	163 (4)
O2—H2⋯N2	0.82	1.83	2.545 (4)	145

Symmetry code: (i) 
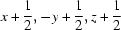
.

**Fig. 1.**
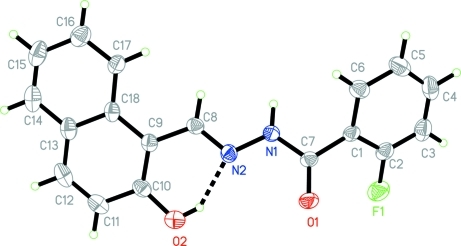
The molecular structure of the title compound, with the numbering scheme and displacement ellipsoids drawn at the 30% probability level. Intramolecular O—H···N hydrogen bond is shown as a dashed line.

**Fig. 2.**
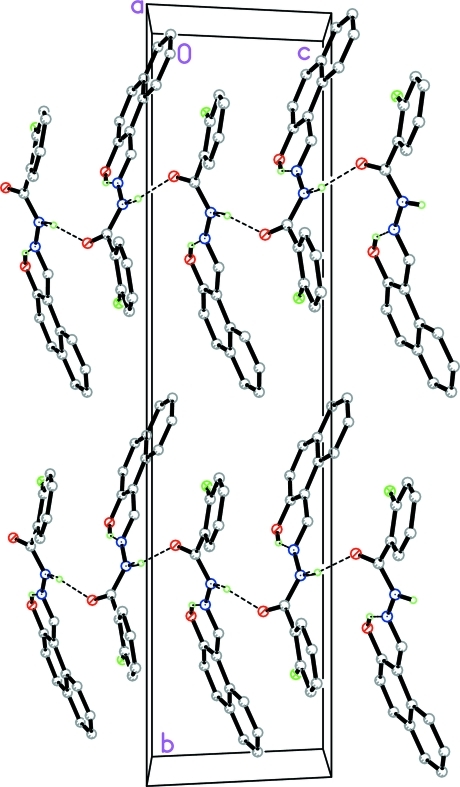
A portion of the crystal packing with intermolecular N—H···O hydrogen-bonds shown by dashed lines. H-atoms not involved in hydrogen bonding omitted for clarity.

## supplementary crystallographic information

### Comment

Benzohydrazide compounds are well known due to their biological activities (El-Sayed *et al.*, 2011; Horiuchi *et al.*, 2009). In addition, benzohydrazide compounds can also been used as versatile ligands in coordination chemistry (El-Dissouky *et al.*, 2010; Zhang *et al.*, 2010). As a contribution to a structural study on hydrazone compounds, we present here the crystal structure of the title compound (I) obtained in the reaction of 2-hydroxy-1-naphthaldehyde with 2-fluorobenzohydrazide in methanol.

In (I) (Fig. 1), the naphthyl mean plane and the benzene ring form a dihedral angle of 31.0 (2)°. The bond distances and angles are within normal ranges (Allen *et al.*, 1987), and agree well with the corresponding bond distances and angles reported for related compounds (Suleiman Gwaram *et al.*, 2010; Liu *et al.*, 2011; Zhou *et al.*, 2011; Meng *et al.*, 2011).

In the crystal, intermolecular N—H···O hydrogen bonds (Table 1) link the molecules into chains (Fig. 2) propagated in [101].

### Experimental

To a methanol solution (20 ml) of 2-hydroxy-1-naphthaldehyde (0.1 mmol, 17.2 mg) and 2-fluorobenzohydrazide (0.1 mmol, 15.4 mg), a few drops of acetic acid were added. The mixture was refluxed for 1 h and then cooled to room temperature. The white crystalline solid was collected by filtration, washed with cold methanol and dried in air. Single crystals, suitable for X-ray diffraction, were obtained by slow evaporation of a methanol solution of the product in air.

### Refinement

The amino H atom was located in a difference Fourier map and was refined with a distance restraint, N—H = 0.90 (1) Å. The C- and O-bound H atoms were positioned geometrically and refined using a riding model, with C—H = 0.93 Å, O—H = 0.82 Å, and with *U*_iso_(H) = 1.2 × *U*_eq_(C) and 1.5 × *U*_eq_(O). In the absence of any significant anomalous scatterers in the molecule, the 1057 Friedel pairs were merged before the final refinement.

### Crystal data

**Table:**

C_18_H_13_FN_2_O_2_	*F*(000) = 640
*M**_r_* = 308.30	*D*_x_ = 1.416 Mg m^−^^3^
Monoclinic, *C**c*	Mo *K*α radiation, λ = 0.71073 Å
*a* = 7.078 (3) Å	Cell parameters from 1565 reflections
*b* = 29.1953 (16) Å	θ = 2.7–24.3°
*c* = 7.3013 (10) Å	µ = 0.10 mm^−^^1^
β = 106.521 (3)°	*T* = 298 K
*V* = 1446.5 (6) Å^3^	Block, colourless
*Z* = 4	0.20 × 0.17 × 0.17 mm

### Data collection

**Table:**

Bruker SMART 1K CCD area-detector diffractometer	2641 independent reflections
Radiation source: fine-focus sealed tube	1777 reflections with *I* > 2σ(*I*)
graphite	*R*_int_ = 0.036
ω scan	θ_max_ = 27.0°, θ_min_ = 2.8°
Absorption correction: multi-scan (*SADABS*; Sheldrick, 1996)	*h* = −9→9
*T*_min_ = 0.980, *T*_max_ = 0.983	*k* = −37→36
4745 measured reflections	*l* = −9→5

### Refinement

**Table:**

Refinement on *F*^2^	Primary atom site location: structure-invariant direct methods
Least-squares matrix: full	Secondary atom site location: difference Fourier map
*R*[*F*^2^ > 2σ(*F*^2^)] = 0.046	Hydrogen site location: inferred from neighbouring sites
*wR*(*F*^2^) = 0.133	H atoms treated by a mixture of independent and constrained refinement
*S* = 1.03	*w* = 1/[σ^2^(*F*_o_^2^) + (0.0679*P*)^2^ + 0.0478*P*] where *P* = (*F*_o_^2^ + 2*F*_c_^2^)/3
2641 reflections	(Δ/σ)_max_ < 0.001
212 parameters	Δρ_max_ = 0.18 e Å^−^^3^
3 restraints	Δρ_min_ = −0.16 e Å^−^^3^

### Special details

**Table:**

Geometry. All e.s.d.'s (except the e.s.d. in the dihedral angle between two l.s. planes) are estimated using the full covariance matrix. The cell e.s.d.'s are taken into account individually in the estimation of e.s.d.'s in distances, angles and torsion angles; correlations between e.s.d.'s in cell parameters are only used when they are defined by crystal symmetry. An approximate (isotropic) treatment of cell e.s.d.'s is used for estimating e.s.d.'s involving l.s. planes.
Refinement. Refinement of *F*^2^ against ALL reflections. The weighted *R*-factor *wR* and goodness of fit *S* are based on *F*^2^, conventional *R*-factors *R* are based on *F*, with *F* set to zero for negative *F*^2^. The threshold expression of *F*^2^ > σ(*F*^2^) is used only for calculating *R*-factors(gt) *etc*. and is not relevant to the choice of reflections for refinement. *R*-factors based on *F*^2^ are statistically about twice as large as those based on *F*, and *R*- factors based on ALL data will be even larger.

### Fractional atomic coordinates and isotropic or equivalent isotropic displacement parameters (Å^2^)

**Table:**

	*x*	*y*	*z*	*U*_iso_*/*U*_eq_	
F1	0.1283 (4)	0.37460 (7)	0.8168 (4)	0.0749 (7)	
N1	0.1961 (4)	0.24093 (9)	0.8406 (4)	0.0472 (7)	
N2	0.0468 (4)	0.20916 (9)	0.8074 (4)	0.0454 (7)	
O1	0.0092 (3)	0.29182 (7)	0.6358 (3)	0.0568 (6)	
O2	−0.3144 (4)	0.18598 (9)	0.7215 (4)	0.0626 (7)	
H2	−0.2211	0.2032	0.7283	0.094*	
C1	0.3335 (4)	0.31244 (10)	0.8066 (4)	0.0406 (7)	
C2	0.3090 (5)	0.35837 (11)	0.8366 (5)	0.0498 (8)	
C3	0.4602 (7)	0.38825 (14)	0.8874 (6)	0.0664 (11)	
H3	0.4377	0.4191	0.9055	0.080*	
C4	0.6445 (7)	0.37250 (16)	0.9116 (6)	0.0723 (13)	
H4	0.7500	0.3927	0.9482	0.087*	
C5	0.6796 (5)	0.32759 (16)	0.8836 (6)	0.0698 (12)	
H5	0.8075	0.3173	0.8989	0.084*	
C6	0.5243 (5)	0.29761 (13)	0.8327 (5)	0.0549 (9)	
H6	0.5480	0.2668	0.8156	0.066*	
C7	0.1631 (4)	0.28143 (10)	0.7509 (4)	0.0412 (7)	
C8	0.0955 (5)	0.17013 (11)	0.8843 (5)	0.0427 (7)	
H8	0.2266	0.1642	0.9488	0.051*	
C9	−0.0507 (4)	0.13518 (11)	0.8723 (4)	0.0417 (7)	
C10	−0.2470 (5)	0.14513 (12)	0.7981 (5)	0.0485 (8)	
C11	−0.3917 (6)	0.11228 (14)	0.7994 (6)	0.0615 (10)	
H11	−0.5245	0.1194	0.7495	0.074*	
C12	−0.3365 (6)	0.07047 (14)	0.8733 (6)	0.0655 (11)	
H12	−0.4334	0.0490	0.8733	0.079*	
C13	−0.1387 (6)	0.05826 (11)	0.9502 (5)	0.0534 (9)	
C14	−0.0837 (7)	0.01541 (13)	1.0315 (6)	0.0691 (11)	
H14	−0.1810	−0.0060	1.0314	0.083*	
C15	0.1059 (8)	0.00411 (13)	1.1100 (6)	0.0718 (12)	
H15	0.1393	−0.0246	1.1645	0.086*	
C16	0.2514 (6)	0.03585 (12)	1.1089 (5)	0.0641 (10)	
H16	0.3832	0.0284	1.1635	0.077*	
C17	0.2030 (5)	0.07762 (10)	1.0289 (5)	0.0505 (8)	
H17	0.3030	0.0982	1.0272	0.061*	
C18	0.0072 (5)	0.09071 (10)	0.9490 (4)	0.0436 (8)	
H1	0.311 (4)	0.2354 (13)	0.931 (4)	0.080*	

### Atomic displacement parameters (Å^2^)

**Table:**

	*U*^11^	*U*^22^	*U*^33^	*U*^12^	*U*^13^	*U*^23^
F1	0.0780 (15)	0.0538 (12)	0.0873 (17)	0.0145 (11)	0.0145 (12)	0.0005 (11)
N1	0.0436 (15)	0.0432 (15)	0.0443 (17)	−0.0058 (11)	−0.0042 (12)	0.0034 (12)
N2	0.0497 (16)	0.0401 (15)	0.0415 (16)	−0.0063 (12)	0.0048 (12)	−0.0037 (12)
O1	0.0516 (14)	0.0480 (12)	0.0549 (16)	0.0006 (10)	−0.0108 (12)	0.0027 (11)
O2	0.0512 (13)	0.0647 (17)	0.0658 (17)	0.0022 (12)	0.0066 (12)	0.0068 (13)
C1	0.0448 (17)	0.0422 (17)	0.0290 (17)	0.0014 (13)	0.0011 (13)	0.0037 (13)
C2	0.059 (2)	0.0475 (19)	0.0366 (19)	0.0014 (17)	0.0041 (16)	0.0039 (15)
C3	0.087 (3)	0.053 (2)	0.049 (2)	−0.018 (2)	0.004 (2)	0.0039 (18)
C4	0.077 (3)	0.082 (3)	0.047 (2)	−0.037 (2)	0.000 (2)	0.014 (2)
C5	0.046 (2)	0.105 (3)	0.055 (2)	−0.009 (2)	0.0088 (18)	0.020 (2)
C6	0.048 (2)	0.069 (2)	0.046 (2)	0.0003 (17)	0.0103 (15)	0.0072 (17)
C7	0.0419 (17)	0.0411 (17)	0.0361 (18)	0.0043 (13)	0.0040 (15)	−0.0010 (14)
C8	0.0414 (16)	0.0457 (18)	0.0379 (18)	−0.0017 (13)	0.0060 (14)	−0.0039 (14)
C9	0.0494 (19)	0.0422 (18)	0.0318 (17)	−0.0057 (14)	0.0087 (14)	−0.0042 (13)
C10	0.0467 (19)	0.059 (2)	0.036 (2)	−0.0062 (15)	0.0065 (15)	−0.0045 (16)
C11	0.047 (2)	0.079 (3)	0.056 (2)	−0.0119 (18)	0.0103 (17)	−0.007 (2)
C12	0.066 (3)	0.075 (3)	0.058 (3)	−0.031 (2)	0.021 (2)	−0.012 (2)
C13	0.072 (2)	0.049 (2)	0.041 (2)	−0.0151 (17)	0.0197 (17)	−0.0073 (16)
C14	0.102 (3)	0.053 (2)	0.056 (3)	−0.024 (2)	0.029 (3)	−0.0059 (19)
C15	0.112 (4)	0.047 (2)	0.055 (3)	−0.005 (2)	0.022 (3)	0.0050 (18)
C16	0.079 (3)	0.057 (2)	0.052 (2)	0.007 (2)	0.0120 (19)	0.0018 (18)
C17	0.059 (2)	0.0412 (17)	0.049 (2)	0.0013 (16)	0.0116 (17)	0.0006 (15)
C18	0.0588 (19)	0.0397 (17)	0.0340 (18)	−0.0087 (14)	0.0159 (15)	−0.0080 (13)

### Geometric parameters (Å, °)

**Table:**

F1—C2	1.332 (4)	C8—C9	1.438 (4)
N1—C7	1.339 (4)	C8—H8	0.9300
N1—N2	1.375 (3)	C9—C10	1.371 (4)
N1—H1	0.905 (10)	C9—C18	1.427 (4)
N2—C8	1.274 (4)	C10—C11	1.405 (5)
O1—C7	1.210 (4)	C11—C12	1.347 (5)
O2—C10	1.346 (4)	C11—H11	0.9300
O2—H2	0.8200	C12—C13	1.398 (5)
C1—C6	1.379 (5)	C12—H12	0.9300
C1—C2	1.378 (4)	C13—C14	1.392 (5)
C1—C7	1.470 (4)	C13—C18	1.403 (4)
C2—C3	1.348 (5)	C14—C15	1.341 (6)
C3—C4	1.347 (6)	C14—H14	0.9300
C3—H3	0.9300	C15—C16	1.387 (6)
C4—C5	1.361 (6)	C15—H15	0.9300
C4—H4	0.9300	C16—C17	1.353 (4)
C5—C6	1.371 (5)	C16—H16	0.9300
C5—H5	0.9300	C17—C18	1.396 (5)
C6—H6	0.9300	C17—H17	0.9300
			
C7—N1—N2	119.4 (2)	C10—C9—C8	120.3 (3)
C7—N1—H1	121 (3)	C18—C9—C8	120.0 (3)
N2—N1—H1	120 (3)	O2—C10—C9	123.5 (3)
C8—N2—N1	115.4 (3)	O2—C10—C11	115.8 (3)
C10—O2—H2	109.5	C9—C10—C11	120.7 (3)
C6—C1—C2	116.5 (3)	C12—C11—C10	119.5 (4)
C6—C1—C7	122.6 (3)	C12—C11—H11	120.2
C2—C1—C7	120.9 (3)	C10—C11—H11	120.2
F1—C2—C3	117.5 (3)	C11—C12—C13	122.3 (3)
F1—C2—C1	119.3 (3)	C11—C12—H12	118.8
C3—C2—C1	123.2 (3)	C13—C12—H12	118.8
C4—C3—C2	118.6 (4)	C14—C13—C12	121.8 (4)
C4—C3—H3	120.7	C14—C13—C18	119.5 (4)
C2—C3—H3	120.7	C12—C13—C18	118.7 (3)
C3—C4—C5	121.4 (4)	C15—C14—C13	121.8 (4)
C3—C4—H4	119.3	C15—C14—H14	119.1
C5—C4—H4	119.3	C13—C14—H14	119.1
C4—C5—C6	119.3 (4)	C14—C15—C16	119.2 (4)
C4—C5—H5	120.3	C14—C15—H15	120.4
C6—C5—H5	120.3	C16—C15—H15	120.4
C5—C6—C1	121.0 (4)	C17—C16—C15	120.5 (4)
C5—C6—H6	119.5	C17—C16—H16	119.8
C1—C6—H6	119.5	C15—C16—H16	119.8
O1—C7—N1	123.9 (3)	C16—C17—C18	121.8 (3)
O1—C7—C1	122.9 (3)	C16—C17—H17	119.1
N1—C7—C1	113.2 (3)	C18—C17—H17	119.1
N2—C8—C9	120.5 (3)	C17—C18—C13	117.2 (3)
N2—C8—H8	119.7	C17—C18—C9	123.6 (3)
C9—C8—H8	119.7	C13—C18—C9	119.1 (3)
C10—C9—C18	119.6 (3)		

### Hydrogen-bond geometry (Å, °)

**Table:**

*D*—H···*A*	*D*—H	H···*A*	*D*···*A*	*D*—H···*A*
N1—H1···O1^i^	0.91 (1)	1.91 (2)	2.785 (3)	163 (4)
O2—H2···N2	0.82	1.83	2.545 (4)	145.

Symmetry codes: (i) *x*+1/2, −*y*+1/2, *z*+1/2.
